# Compression Devices as an Alternative Approach After Unsuccessful Ring Removal Attempts

**DOI:** 10.7759/cureus.22068

**Published:** 2022-02-09

**Authors:** Landon Sayler, Constance LeBlanc

**Affiliations:** 1 Family Medicine, Dalhousie University, Halifax, CAN; 2 Emergency Medicine, Dalhousie University, Halifax, CAN

**Keywords:** ring removal, compression device, stuck ring, ring, finger, digit trauma, finger trauma, ring preservation, patient comfort, emergency

## Abstract

Removal of a stuck ring is a medical problem sufficiently common for most practitioners to have an approach. However, there is limited consensus on the best approach to care. Commonly used approaches include lubricant, localized cooling, various forms of finger wrapping, attempts at ring and finger manipulation, and finally cutting or breaking the ring for removal. Each approach has its limitations and potential complications. Rings can also have sentimental value to the patient and destroying or damaging a ring may not be acceptable to the patient.

This case report presents an approach to ring removal using a compression device (CD) that successfully allowed medical professionals to remove an intact ring from an injured finger. Several unsuccessful attempts at removal were made before using this CD.

## Introduction

Due to the variety of presentations, ring removal can be challenging for healthcare providers with patients typically presenting in emergency contexts. A new approach to ring removal involves using a compression device (CD) to reduce the edema and overall size of the affected finger before attempting removal [1]. Several compression techniques have been used to remove rings ranging from the application of strings or elastics to tourniquets [2-5]. This novel device has yet to be included in formal recommendations and algorithms for ring removal with other compression techniques [6-7]. This represents a knowledge to practice gap [8]. In our opinion, based on regular use, this compression technique presents a valuable option for the removal of stuck rings.

Some concerns with commonly used traditional approaches to ring removal is that these approaches can cause additional digit trauma (tissue injury from tight wraps, trauma from ring cutting, or lacerations from sharp edges following ring cutting) and can result in significant damage to the ring [5,9-11]. To further complicate removal, modern ring materials and designs can be challenging to cut with cutting devices [9-11]. Also, sometimes missing from the discussion is the sentimental value that jewellery can hold and the loss a patient experiences when jewellery is damaged or destroyed in the process of medical care for their digit [5-6,12-13].

There have been prior case series in the emergency medicine literature describing the benefits of using CDs for ring removal [1]. We report a case of successful CD use after having exhausted other approaches to ring removal without cutting the ring.

## Case presentation

A 63-year-old female presented to a community emergency department 10 days after sustaining an injury to the ring finger on her right hand. Initially, the patient thought the injury to be minor. However, the injured finger became increasingly edematous and painful. Initial attempts were made to remove the ring at home by applying cold packs and lubricants without success, prompting her to seek care. The ring had last been removed approximately six months prior and was a custom piece of jewellery of significant sentimental value to the patient.

**Figure 1:**
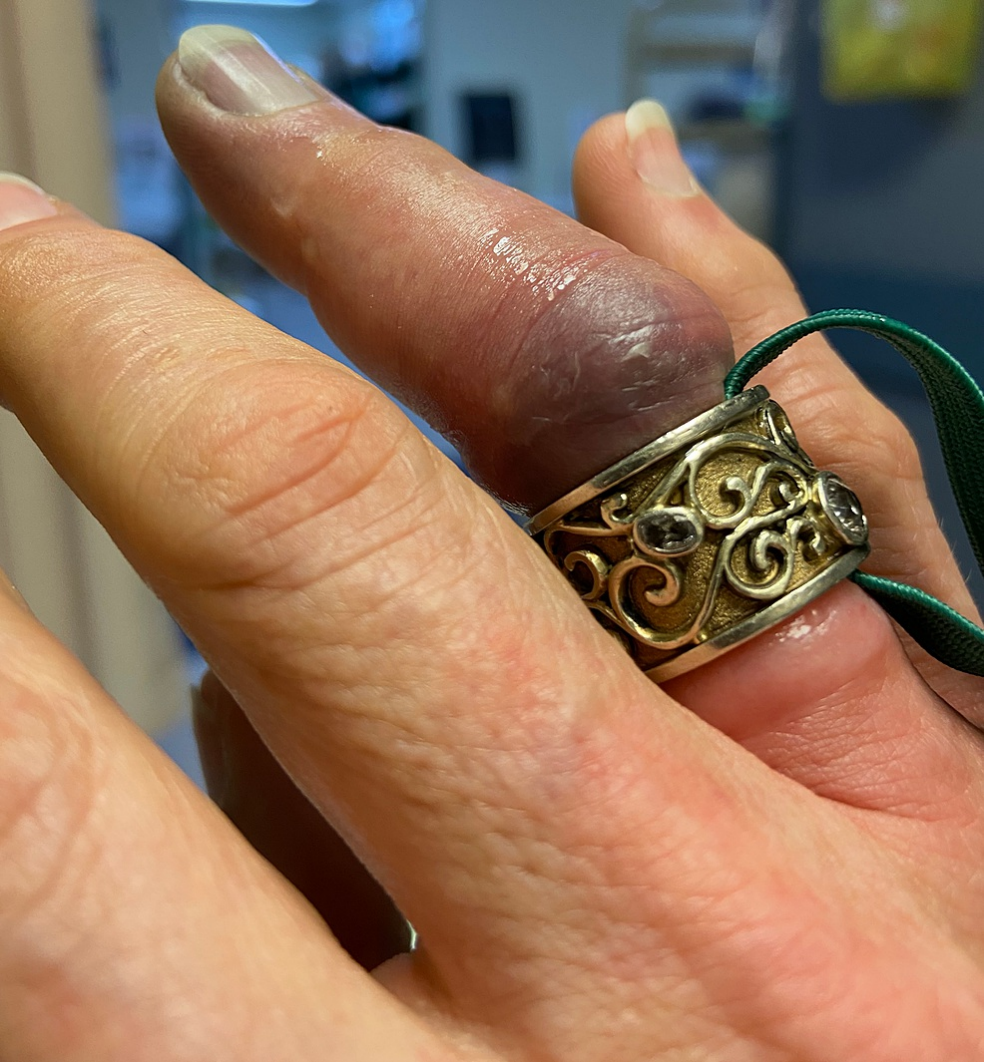
Patient presentation following multiple failed attempts using a string wrap

The clinicians’ first removal attempt involved applying cold packs and lubricants followed by physical manipulation. This approach failed and caused discomfort to the patient. Next, a finger wrap was performed using the elastic strap from an oxygen mask. The tightly wound wrap worsened the edema and the patient experienced increased discomfort. A metacarpal block was completed and three additional attempts were made using finger wrap techniques, all unsuccessful. Due to the increased swelling and bruising (Figure 1), the decision was made to cut the ring. First, a manual ring cutter was used. However, the degree of finger edema together with the thickness of the ring made this option both painful and technically challenging. Next, a Dremel tool was obtained. However, due to the ring material and design, this tool skipped repeatedly posing a risk to both operator and patient. Although the exact composition of this ring was unknown, this is representative of many cases of ring removal.

**Figure 2:**
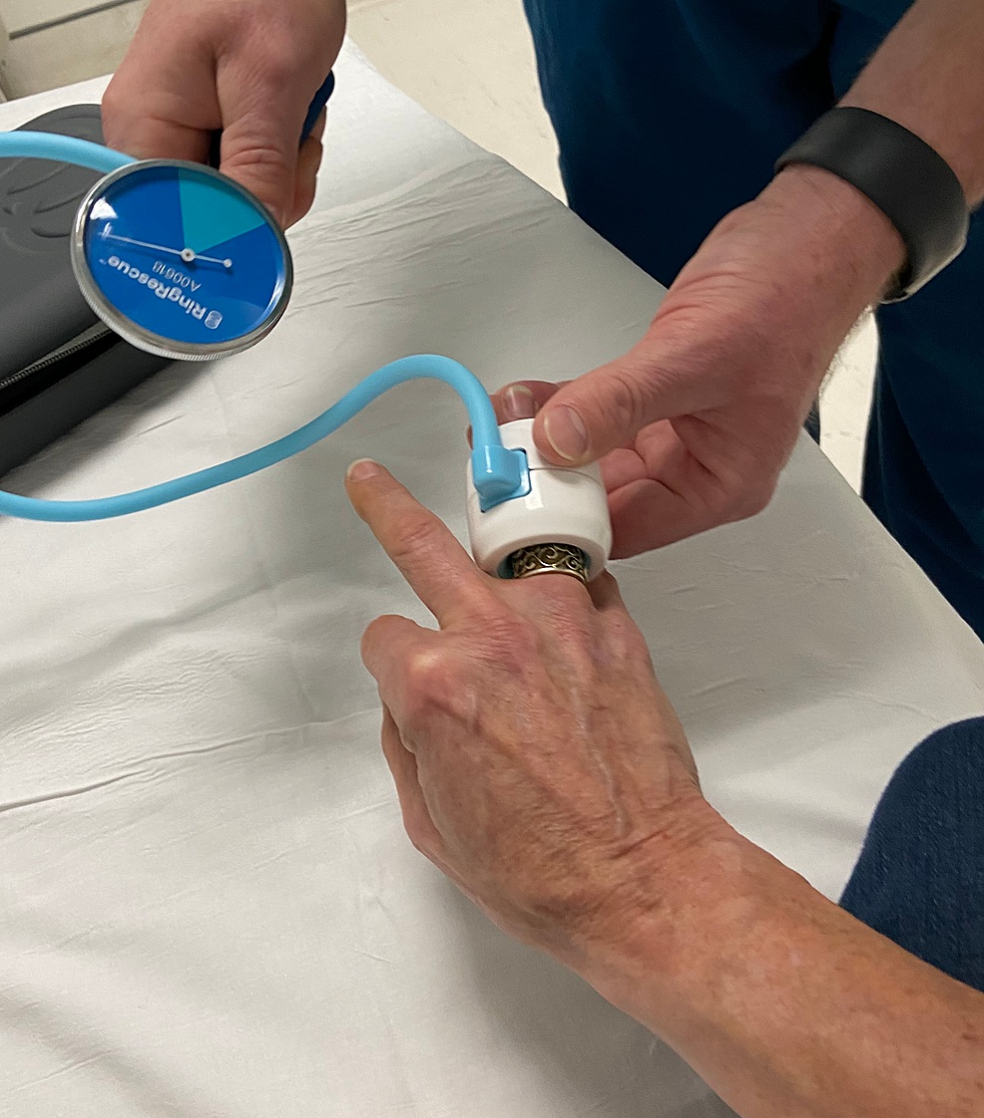
Compression device being applied following transfer to alternative facility

The patient was transferred to another facility where providers were experienced with a CD. The physician first completed a clinical assessment of the injured finger, including an inspection of the ring and x-ray imaging to further establish the extent of the finger injury. Based on these data, it was decided that the use of a CD (Ring Rescue Compression Device, Ring Rescue Inc., Canada) was appropriate. A digital block was performed (anaesthesia from the earlier block had since worn off), and the CD was used according to manufacturer guidelines (Figure 2).

**Figure 3:**
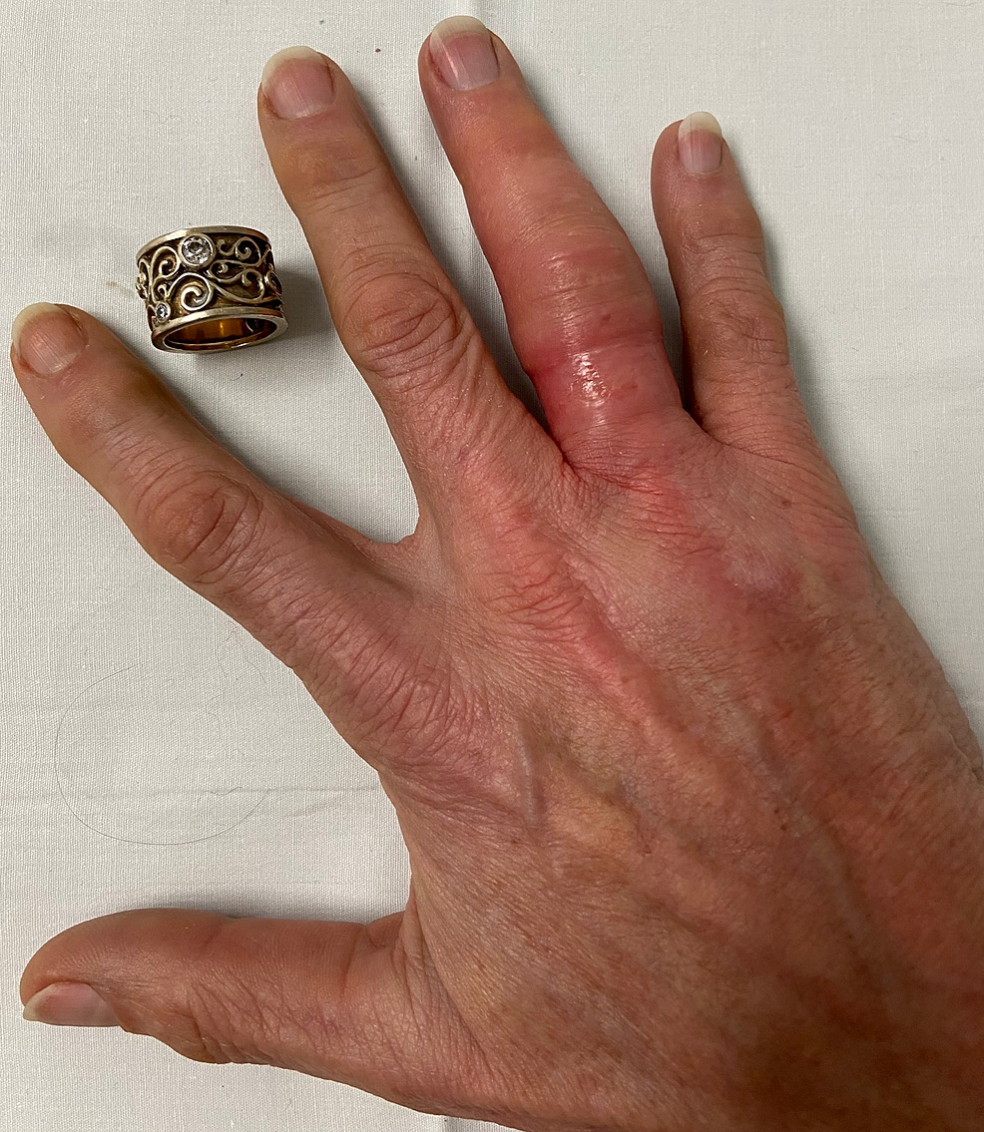
Patient presentation immediately after successful ring removal using a compression device

Three rounds of finger compression were necessary before the finger size reduction was felt to be amenable for proceeding with ring removal. Lubrication was applied and ring manipulation was performed, resulting in successful ring removal (Figure 3). Throughout the procedure, the patient reported minimal discomfort and was relieved with the intact removal of her ring.

## Discussion

This case report demonstrates the potential benefit of using a CD for ring removal when indicated. Fortunately for this patient, CD removal was successful when initial removal approaches were not.

The need for ring removal can result from processes that are systemic, regional, or localized, and can occur acutely or over days to years. Due to the variety of presentations, it is important to assess the cause of a ring becoming stuck [6-7], and like any tool, CDs must be applied only when safe. As with all ring removal techniques, the provider ultimately removes the ring, making proper technique with any tool, and often the integration of several techniques which are important for success. Successful ring removal is more likely once an edematous finger has been compressed and has a reduced diameter, which is the role of the CD.

Additional benefits to using a CD is the potential for a less traumatic patient experience as well as preservation of a ring which may have sentimental value [6]. Although challenging to quantify, these two metrics are not inconsequential and often the provider-patient discussion is focused solely on whether the ring can be removed and the digit preserved [5-7,10-11]. Patient comfort and ring preservation are significant metrics. Asher et al. recognize this when they state that any future algorithms for ring removal should consider patient input when selecting a removal strategy [6].

## Conclusions

In this case, a CD provided an approach for ring removal that might be considered when other methods fail. A randomized controlled trial or significant case series of patients presenting to the emergency department with stuck rings may support the adoption of this approach earlier in the process of care or as a rescue device when other methods have failed. Currently, there is insufficient research to indicate how best to integrate this device into patient care.

There are multiple approaches to finger compression when a patient presents with a stuck ring, which includes strings or elastic wraps, tourniquets, and the use of a CD. The goal of CD is to decrease finger edema and ultimately finger diameter so that a stuck ring can be removed. An approach that causes minimal additional finger trauma is preferential. A compression device is a novel approach to stuck-ring removal that can integrate compression with other removal techniques such as lubrication, caterpillar, two-thread or two-rubber-band technique, along with others. At present there is only one CD kit that is commercially available, which is the device that was used in this case. The only contraindications to CD use is a lack of knowledge or skill in operating the device which could result in additional patient injury.

More research needs to be done to compare the multiple ring removal options and to improve our knowledge in this area. Ideally, an algorithmic approach to ring removal based upon patient presentation, concomitant injuries and conditions, current best-evidence, and patient preferences will support providers to meet this important patient need.
